# Ultraviolet Leaks from CFLs

**DOI:** 10.1289/ehp.120-a387

**Published:** 2012-10-01

**Authors:** Wendee Nicole

**Affiliations:** Wendee Nicole, based in Houston, TX, has written for *Nature*, *Scientific American*, *National Wildlife*, and other magazines.

With growing concern over energy use, much of the developed world has adopted compact fluorescent lamps (CFLs), which use 25–80% less energy and can last 3–25 times longer than regular incandescent bulbs.^1^ A new study suggests that certain elements of these bulbs might be improved for safer use.^2^

Investigators measured ultraviolet (UV) radiation emissions from nine commercially available CFLs and observed cracks in the phosphor coating on each bulb that might allow UV leaks. “Phosphor is very rigid, so it’s not surprising it would crack [when applied to a CFL’s tight coils],” says coauthor Miriam Rafailovich, distinguished professor of materials science at the State University of New York at Stony Brook. She says long, straight fluorescent tubes don’t have these cracks.

The team exposed healthy human keratinocytes and dermal fibroblasts to the CFL with the highest UV emissions at a distance of 2.5 cm for 2 hours at a time. They also tested CFL exposure combined with titanium dioxide (TiO_2_) nanoparticles, a catalyst. CFL exposure was associated with a slight increase in the formation of reactive oxygen species (ROS) in both cell types, reduced mitochondrial activity and cell proliferation in both cell types, and reduced migration velocity and collagen contraction in fibroblasts. These outcomes were greater in combined CFL/TiO_2_ exposure scenarios.

But in contrast to media depictions of “skin-frying” CFLs, researchers are reluctant to draw conclusions about consumer risk on the basis of these findings. “The UV measurement procedures are not described, so one cannot evaluate the data,” says Mats-Olof Mattsson, a cell biology professor at the Austrian Institute of Technology. The authors also reported higher UV emissions than other studies have found^3^^,^^4^^,^^5^ and did not follow international measurement standards,^6^ he adds.

*In vitro* studies have limitations for assessing CFL impacts on skin because the intensity of light reaching the cells within skin is much less than the light intensity at the skin surface. Furthermore, says Harry Moseley, a photobiology professor at the University of Dundee, “Work carried out *in vitro* can be helpful to show the direct effect of UV radiation on the cells, [but] it doesn’t tell us how the body deals with any damage to the cells.”

Nevertheless, the study results are not inconsistent with published research.^5^ “When we have exposed people to [CFL] light, sensitive patients do get a sunburn, and a small proportion of normal people get a mild sunburn,” he says.

**Figure:**
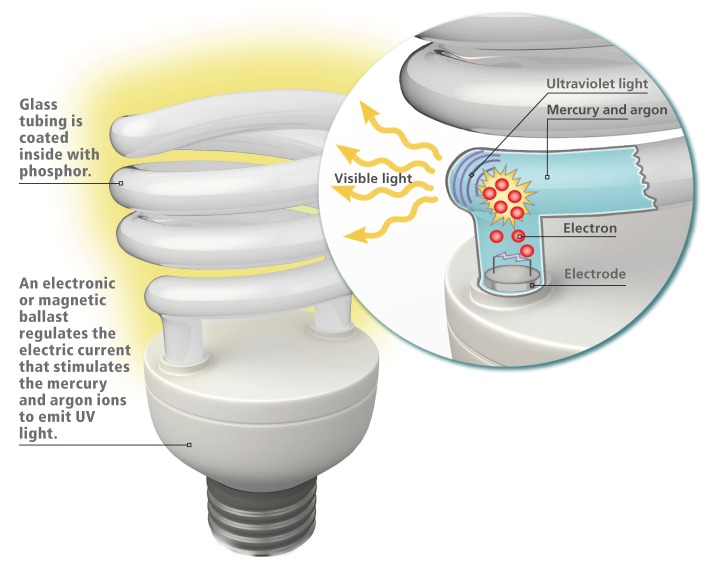
Inside a CFL, argon gas and mercury vapor are stimulated by the flow of electricity, producing UV light. This light hits the phosphor coating painted inside the bulb and causes it to fluoresce. The phosphor blocks most but not all of the UV light, which is radiated along with visible light. Joseph Tart/EHP

The Stony Brook study comes on the heels of several recent reports addressing artificial light. In 2008 Europe’s Scientific Committee on Emerging and Newly Identified Health Risks (SCENIHR) reviewed the literature on the topic and concluded that the flicker and UV emissions from CFLs could adversely affect sensitive individuals with epilepsy, migraine headaches, eye diseases, and skin diseases affected by light.^7^ Numerous medications and personal care products—including antibiotics, antidepressants, diuretics, antipsychotics, and certain cosmetics—can render people hypersensitive to UV light.

In 2012 SCENIHR again addressed the issue of artificial light exposure with an eye to the general public and determined no studies had yet evaluated CFL-specific links to adverse health effects. However, the committee did find substantial evidence linking “single-envelope” CFLs—those with the bare spiral tube showing—to aggravation of chronic actinic dermatitis, solar urticaria, lupus erythematosus, and photosensitive eye conditions. The committee concluded that CFLs pose little short-term health risk for people of normal sensitivity but recommended that all people should avoid using CFLs for close-range desk or task lighting.^6^

“A real problem for the public health aspect is that we have really insufficient knowledge about the actual exposure [to UV radiation],” says Mattsson, who chaired the SCENIHR in 2012. “Emissions are not the same as knowing the exposure.”

That’s an important distinction, says Brian Pollack, an assistant professor of dermatology and pathology at Emory University, because “UV radiation is carcinogenic. The bottom line is if these [bulbs] are emitting UV radiation of any amount, it needs to be defined, and it needs to be prevented.”

UV radiation from CFLs can often, but not always, be avoided by purchasing “double-envelope” bulbs in which the spiral tube is enclosed in a glass or polycarbonate cover resembling a standard incandescent bulb.^3^ The U.S. Food and Drug Administration^8^ and Health Canada^9^ advise that single-envelope CFLs should not be used at distances closer than about one foot.
